# War trauma and torture experiences reported during public health screening of newly resettled Karen refugees: a qualitative study

**DOI:** 10.1186/s12914-015-0046-y

**Published:** 2015-04-08

**Authors:** Tonya L Cook, Patricia J Shannon, Gregory A Vinson, James P Letts, Ehtaw Dwee

**Affiliations:** School of Social Work, University of Minnesota, 1404 Gortner Avenue, St. Paul, MN 55108 USA; The Center for Victims of Torture, 649 Dayton Avenue, St. Paul, MN 55104 USA; HealthEast Care System, St. Paul, MN USA; St. Paul, MN USA

**Keywords:** Karen refugees, Burma, Torture, War trauma, HURIDOCS, Qualitative

## Abstract

**Background:**

Karen refugees have suffered traumatic experiences that affect their physical and mental health in resettlement. The United States Centers for Disease Control and Prevention recommends assessing traumatic histories and mental health symptoms during initial public health screening. This article reports the traumatic experiences that Karen refugees were able to describe during a short screening and contributes knowledge to existing human rights documentation systems.

**Methods:**

Four semi-structured and open-ended items asked about lifetime experiences of war trauma and torture. Interviews were completed with adult, Karen refugees during their initial public health screening. Experiences of war trauma and torture were coded using the extensive Human Rights Information and Documentation (HURIDOCS) Micro-thesauri coding system. Additional codes were created to describe experiences not captured by existing codes.

**Results:**

Over 85% of 179 Karen people interviewed experienced life-threatening war trauma. All participants who reported war trauma or torture stories were able to describe at least one event. New war trauma codes proposed include: widespread community fear, systematic destruction/burning of house or village, exposure to dead bodies, orphaned in the context of war, injury caused by a landmine, fear of Thai police or deportation from Thailand, and harm or killings in the context of war. New torture codes include: forced portering; forced to be a human landmine sweep; forced to be a soldier, including child soldier; forced contact with a dead body; and removal of the eyes.

**Conclusion:**

Karen refugees were able to report traumatic experiences in the context of a brief health screening. The findings confirm existing reports of human rights violations against Karen people and suggest that additional codes be added to the HURIDOCS Micro-thesauri system that is used by torture treatment centers. Understanding the nature of traumatic experiences of this group is important for health providers working with resettled Karen refugees in their countries of resettlement. Health providers may need specialized training to understand the traumatic histories of this new refugee group, learn how to initiate conversations about trauma and its impact on health, and make appropriate mental health referrals in the context of a brief public health screening.

## Background

Approximately 400,000 Karen people are internally displaced and 128,000 refugees from Burma are living in camps on the Thailand-Burma border [1,2]. The majority (79%) of refugees living in these camps are of Karen ethnicity [2]. Karen people are one of seven minority ethnic groups in Burma who have experienced decades of political violence by the Burmese military [3]. Since 2006, the United States has resettled over 75,000 refugees from these camps [4]. Several studies have found significant associations between experiences of refugee trauma and poor physical and mental health outcomes [5,6]. In response to mounting evidence documenting the psychological impact of trauma on refugees, the United States (U.S.) Centers for Disease Control and Prevention (CDC) published recommendations for mental health screening during the domestic medical examination of newly arrived refugees [7]. Their recommendations include: providers being knowledgeable about the histories of the populations they serve, inquiring about patients’ exposure to trauma, and screening refugees for mental health symptoms.

The data in this report are part of a project to develop culturally grounded processes and tools for identifying newly arrived refugees who have experienced trauma and who may need further mental health assessment. We previously reported rates of war trauma, torture and associated mental health symptoms in newly arrived Karen refugees [8]. In this article we report the traumatic experiences that Karen refugees were willing and able to describe in the context of a short mental health screening during their initial, domestic medical examination. Knowledge of the war trauma and torture experiences of Karen people also contributes to existing human rights documentation systems that are widely used by torture treatment centers [9,10].

Karen resistance groups have been engaged in an armed struggle against the Burmese government for an independent Karen State since 1949, the year after Burma gained its own independence [5]. The Burmese government and its military, known as the *Tatmadaw*, have used fear and violence to terrorize Karen and other ethnic minority groups in Burma and to repress resistance, including directly targeting civilian men, women and children [11]. Local and international human rights organizations regularly and meticulously document human rights abuses by the Burmese military, which include torture, extrajudicial killing, destruction of villages, forced displacements and hiding in the jungle, forced labor, extortion, land confiscation, and other violence [12-15]. Reports by Karen and Shan women’s groups have specifically documented women’s vulnerability to systematic and brutal rape, including gang rape, by Burmese military soldiers who use rape as a tactic to terrorize, shame, and subjugate ethnic communities [16,17].

The constant demand for labor and forced porters by the Burmese military as it operates in remote jungle areas affects all aspects of life for ethnic minorities, causing food insecurity, disrupted educations, decreased health, and increased displacement as villagers flee advancing military groups [18]. Forced labor may be the most widespread abuse suffered by Karen people today [18]. Serious human rights violations occur in the context of forced portering, including rape, being used as a human shield, or being forced to do human landmine sweeping in frontline combat operations, also known as “atrocity demining” [16,19]. Mortality surveys of internally displaced Karen and other ethnic minorities inside Burma have also documented the use of forced labor and forced portering by the Burmese military, as well as deaths due to landmines or other violent causes, landmine injuries, forced relocation, and burning of entire villages [20-22]. Resource and security constraints and time-limited reporting periods limited the scope of data that could be collected in these studies.

Only two academic studies have interviewed Karen refugees in resettlement about their traumatic experiences [8,23]. One study of 70 refugees from Burma, primarily Karen, who were resettled to Australia used a 17-item version of the Harvard Trauma Questionnaire (HTQ) and reported that nearly 30% had experienced torture and 46% had witnessed torture [23]. Pre-migration traumas reported included lack of food or water (76%), lack of shelter (69%), combat situations (58%), and ill health without access to medical care (56%). Participants also reported witnessing the serious injury (65%) or rape (33%) of others. Although this study documents some types of torture, the authors identified ‘forced labour’ as a significant omission, noting that it was reported frequently, despite not being a part of the questionnaire [23]. In our sample of 179 Karen refugees, 27.4% experienced or witnessed torture and 51.4% reported the torture of family members. More than 85% of Karen participants reported experiencing war trauma [8].

One study of 495 refugees, mostly Karenni, living in camps on the Thailand-Burma border utilized information from key informant interviews to adapt the HTQ to 31-items that fit the traumatic events experienced by Karenni refugees in Thailand [24]. Comparing this adapted HTQ to the 17-item used by Schweitzer and colleagues supports the importance of adapting existing instruments to fit the experiences of different groups [25]. Seven of the 10 most commonly reported events in the adapted version are not included in the 17-item HTQ and include: hiding in the jungle (79.4%), forced relocation (67.5%), lost property or belongings (66.3%), forced labor (50.5%), destruction/burning of crops (48.5%), destruction/burning of houses (48.1%), and fear of deportation from Thailand (47.7%) [24]. Additionally, over 34% experienced forced portering. These results indicate that the experiences of refugees from Burma may be different from those of previous refugee groups.

Academic studies corroborating human rights reports have been limited by methodological constraints, including the use of quantitative measures and safety constraints. Quantitative measures adopt a priori assumptions about the range of conflict victimization experiences that may not fit the experiences of this population [26]. By using semi-structured and open-ended questions, asking about lifetime experiences, and conducting interviews in the safety of third country resettlement, this academic study adds additional understanding about a broader range of human rights violations experienced by Karen people in the context of a complex and protracted conflict.

One universal system widely accepted by torture treatment programs for documenting and classifying war trauma and torture experiences is the Human Rights Information and Documentation Systems, International (HURIDOCS) Micro-Thesauri [9,10]. HURIDOCS is an informal and open network of human rights organizations whose purpose is to develop international standards and work towards a universal system for documenting and communicating about human rights information [27]. In collaboration with other human rights groups, HURIDOCS developed the Events Standard Formats methodology that includes a Micro-thesauri, a collection of terminology lists useful for documenting human rights violations. We used this existing coding system in our data analysis to examine its utility, highlight the experiences of Karen refugees that are not included in the current lists, and to be maximally useful to human rights organizations and others working in the field.

Understanding the full extent of the traumatic experiences of Karen refugees is important to addressing associated health and mental health conditions. While resettlement to a third country is challenging in itself, research indicates that refugees exposed to torture and war trauma may face additional physical and mental health-related barriers to successful resettlement that must be addressed [5,28,29]. The two studies that interviewed Karen refugees in resettlement also documented mental health symptoms. The study of 70 Karen refugees resettled to Australia used the HSCL-37 and found the following rates of PTSD (9%), anxiety (20%), depression (36%), and somatization symptoms (37%) [23]. In our larger sample of 179, being a torture survivor (27.4%) was significantly related to total distress, posttraumatic stress, and somatic item scores [8].

Emerging evidence also demonstrates a link between exposure to traumatic events and development of chronic disease and poor health outcomes. These include morbidity and mortality related to heart disease, hypertension, metabolic syndrome, diabetes, musculoskeletal conditions, chronic pain, and other diseases [30,31]. These effects on health are independent of psychological conditions and appear to be driven by changes in physiology caused by recurrent and severe trauma exposure [32]. Studies of Karen people have documented associations between conflict victimization or politically motivated oppression and pregnancy complications [33], intimate partner violence [34], and prevalence of infectious disease [35]. The U.S. Centers for Disease Control report that possible health conditions of Karen refugees include: tuberculosis, HIV/AIDS, diabetes mellitus, end-stage renal disease, cancer, Hepatitis B, and substance abuse [36]. Early identification of refugees who have experienced trauma and associated health and mental health symptoms is important because refugees in the United States have access to public health care within the first eight months of their resettlement [37].

## Methods

### Study design and procedures

The methods of this study have been previously described [8] and are summarized here. Between May 2011 and May 2013, Karen refugees age 18 or over were invited by clinic staff to participate in this study during their initial health screening, which took place in a primary care clinic. Two trained research assistants and three trained, professional interpreters reviewed health privacy laws, obtained informed consent, collected demographics, and completed a mental health screening interview that also asked about their experiences of war trauma and torture. All forms went through a rigorous translation, back translation, and reconciliation process and copies of all signed forms were given to participants in English and Karen. Two participants declined to participate in the study, 2 interviews were discontinued due to distress, and 179 participants completed the full screening. This manuscript complies with RATS guidelines for reporting on qualitative studies.

An introduction normalized that trauma is a common experience of refugees, then four semi-structured items asked retrospectively about lifetime experiences of primary and secondary war trauma and torture: (a) In your life, have you ever been harmed or threatened by the following: government, police, military or rebel soldiers, or other? If yes, what was it? (b) Has any of your family ever been harmed or threatened by the following: government, police, military or rebel soldiers, or other? If yes, what was it? (c) Some people in your situation have experienced torture. Has that ever happened to you? If yes, what was it? and (d) Has anyone in your family been tortured? If yes, what was it? Previous research has identified these questions as effective for eliciting patients’ war trauma and torture histories in primary care settings [38].

If participants responded in the negative to the first question they were asked, “why did you leave your home country?” Focused follow up questions were used to determine whether the participant was a survivor of torture according to the US definition of torture:

an act committed by a person acting under the color of law specifically intended to inflict severe physical or mental pain or suffering (other than pain or suffering incidental to lawful sanctions) upon another person within his custody or physical control [39].

An example is asking the participant to clarify who perpetrated the torture in order to determine whether it was someone acting under the color of law. It took approximately 5-7 minutes to elicit participants’ responses to these questions. Responses were recorded verbatim, de-identified, and entered into a spreadsheet for coding.

By using semi-structured and open-ended questions, we did not limit the types of war trauma and torture experiences participants could report. Also, participants were allowed to report any violation experienced in any country. We did not attempt to capture an exhaustive list of all war trauma and torture experiences or to document the frequencies of types reported.

It is possible that trauma experiences affected some participants’ ability to recall their experiences, as is a feature of posttraumatic stress [40]. However, all participants that identified as trauma survivors in this study were able to describe at least one event. Many participants described experiencing multiple, prolonged traumas over their life course.

The following steps were taken to prevent re-traumatization. Participants were informed of the purpose of the study and nature of the questions and asked if they would like to learn more about participating. If yes, they were read a consent form in Karen that described risks of participating in the study and the personal nature of the questions, gave permission to stop the interview at any time for any reason, and participants were given a chance to ask questions before agreeing to participate in the study. Research assistants were trained Masters or Ph.D. level social work students with previous experience interviewing refugees who had access to medical personnel at all times. The assessor team stopped two screenings due to distress and referred the participants immediately to their medical provider. All participants saw a medical provider following the screening. The medical provider received a copy of the completed screening forms and initiated further assessment, treatment, or referrals for any identified health concerns.

As expected, it was difficult for survivors to talk about traumatic experiences. In this study, many participants became tearful. However, when given the option to stop the interview, almost every participant stated that they wanted to continue, for several reasons. First, the goals of the study were explained well, and participants believed the information was relevant to their healthcare and would also help their community. Second, for most participants, this was the first time they had a chance to have their experiences of trauma validated. Several factors also helped participants feel comfortable sharing their experiences. The interviews took place in a clinic that is well-known in the local Karen community and has a reputation for providing good care, the main interpreter was familiar and trusted by most participants, and the interviewers maintained a kind and warm demeanor. The majority of participants appeared relieved at the end of the interviews, and a few independently reported feeling as if a heavy load had been lifted.

### Role of researchers

The principle investigators on this study have extensive experience conducting research and working clinically with refugee populations at the Center for Victims of Torture and a primary care clinic that has a large Karen patient population. The Karen cultural consultant is an independent, professional interpreter and Karen community liaison and served as a translator, interpreter and cultural consultant on this project. He participated in extensive, videotaped training on interviewing and gave cultural guidance about the screening questions and process.

When working cross-culturally, cultural consultants enhance the validity of qualitative data by verifying that researchers understand accurately the meaning of what is being interpreted [41]. When necessary, our cultural consultant provided cultural and historical context for participants’ stories, such as explaining terms like “black zone,” a dangerous “free-fire” zone controlled by the Burmese military, or “Kawthoolei,” a Karen word used to refer to the Karen National Union. This allowed interviewers to understand brief stories without having to ask participants for additional context. Methodologically, cultural consultants are commonly used in qualitative research to enhance the trustworthiness of findings [42]. As a strategy for cultural verification, the consultant reviewed, gave feedback on, and approved the final analysis and manuscript.

### Ethical approval

Ethical approval for this study was obtained from the Institutional Review Boards of the University of Minnesota and the HealthEast Care System.

### Participants

All participants (*N* = 179) were of Karen ethnicity and arrived to the U.S. with refugee status. The sample was evenly split between men (51%) and women (49%). The mean age was 35 (SD = 14.6), and participants had been in the US for an average of 37 days (SD = 23) at the time of interview. Participants came from the following refugee camps: Mae La Oon (31%), Mae Ra Ma Luang (Mae Ra Moo) (20%), Mae La (Beh Klaw) (18%), Nu Po (12%), Umpiem Mai (10%), Ban Don Yang (6%), and Tham Hin (1%). Two people arrived from urban areas in Malaysia (1%). The average number of years participants lived in the refugee camps was 13 (SD = 5), and 66% reported primary school or none as their highest level of education. Only 16% reported that their entire family was in the U.S. and most reported having family members who were still in Burma or Thailand, including parents (37%), children (22%), spouse (6%), and other (61%). Participants spoke the following languages: S’gaw Karen (97%), Burmese (16%), Pwo Karen (16%), English (12%), Thai (9%) and other (2%).

### Analysis

We used a two-step coding process for coding types of war trauma and torture experiences described by participants. A trained research assistant coded all experiences using existing codes found in the “Methods of Violence” and “Type of Acts” sections of the Human Rights Information and Documentation Systems, International (HURIDOCS) Micro-thesauri [27]. Initial coding was checked and questions were negotiated by a Principle Investigator who has more than 10 years of experience working with torture survivors. While the Micro-thesauri lists are extensive, the coding system is meant to be adapted and allows for the addition of new codes as they arise organically in local settings. Additional codes were created to describe experiences that did not fit within the existing HURIDOCS codes. Responses were subsequently categorized by primary and secondary torture survivors according to how the U.S. defines torture, and by primary and secondary war trauma survivors. War trauma is exposure to an extremely traumatic event in the context of war, defined as:

direct personal experience of an event that involves actual or threatened death or serious injury, or other threat to one’s physical integrity; or witnessing an event that involves death, injury, or threat to the physical integrity of another person; or learning about unexpected or violent death, serious harm, or threat of death or injury experienced by a family member or other close associate [43].

Primary torture survivors experienced torture directly or witnessed the torture of family members. Secondary torture survivors reported the experiences of a family member, which they did not witness.

## Results

All HURIDOCS Micro-thesauri codes that described the primary and secondary war trauma experiences reported by participants are listed in Table 1. All codes used to classify primary and secondary torture experiences are listed in Table 2. In this section, we focus on describing the new war trauma codes that emerged from this study to justify the creation of additional codes and highlight their importance to understanding the Karen experience. Next, we describe new torture codes that emerged in this study. Occasionally, we have removed specific ages or changed a few words in a participant’s description, and bracketed these changes, in order to protect confidentiality.

All acts of war trauma in this study were reported as perpetrated by Burmese soldiers, with the exception of stories under the category of *fear of Thai police or deportation from Thailand* and two participants who reported violence perpetuated by Democratic Karen Buddhist Army (DKBA) soldiers. All acts of torture in this study were reported as perpetrated by Burmese soldiers, with three exceptions. One participant reported being raped by a Karen officer in Thailand, and two others reported being beaten by Thai police.

### Primary and secondary war trauma experiences

Over 85% of Karen people interviewed experienced life-threatening war trauma in instances where they were not specifically targeted for torture [8]. Participants described 29 different types of war trauma, reported in Table 1. Twenty two existing HURIDOCS Micro-thesauri codes were used and 7 new codes were created to describe additional experiences that participants reported. New codes include: *widespread community fear, systematic destruction/burning of house or village, exposure to dead bodies, orphaned in the context of war, injury caused by landmine, fear of Thai police or deportation from Thailand, and harm or killings in the context of war*, and are described further.

**Table 1:** **Types of war trauma reported by primary and secondary survivors**

**Types**	**Primary survivors**	**Secondary survivors**
Threats (against the victim, victim's family, or friends)	x	x
Collective punishments other than mass roundup or curfew	x	x
Displacement	x	x
Restriction on travel	x	x
Restriction on the practice of religion	x	x
Looting; theft	x	x
Confiscation of property	x	x
Extortion	x	x
Destruction of property	x	x
Land expropriation	x	x
Direct actions which violate the right to adequate housing	x	x
Direct actions which violate the right to adequate food	x	x
Denial of appropriate health treatment	x	x
Direct actions which violate the right to education	x	x
Banning/restrictions on the use of a particular language	x	x
Banning/restrictions on certain cultural practices	x	x
Shooting as a method of indiscriminate attack	x	x
Bombing as a method of indiscriminate attack	x	x
Arson as a method of indiscriminate attack	x	x
Other methods of indiscriminate attack	x	x
Widespread community fear^a^	x	x
Systematic destruction/burning of house or village^a^	x	x
Exposure to dead bodies^a^	x	x
Orphaned in the context of war^a^	x	x
Injury caused by landmine^a^	x	x
Fear of Thai police or deportation from Thailand^a^	x	x
Harm or killing in the context of war^a^	x	x
Death of a noncombatant in a crossfire		x
Killing in indiscriminate attacks such as bombing		x

^a^Codes created in addition to existing HURIDOCS Micro-thesauri codes.

#### Widespread community fear

*Widespread community fear* was frequently reported by participants. It is clear that participants often experienced sudden attacks on their homes and villages by the Burmese military, multiple and prolonged displacements, deprivation and sickness during displacement, and living in a constant state of fear and insecurity. These seem to be particularly characteristic of the experiences of Karen refugees*.* One participant described widespread community fear in this way:

*One night I heard gun shots. Burmese soldiers were already in front of my house. I took my baby and ran. I heard people crying. This was the day that my little sister was shot and we left that village. After that we were always running.*

Other participants described widespread community fear in these ways: *“Burmese soldiers came to my village. We had to run from place to place among the mortar shells. We were never safe [and] always scared;” “like small chicken, we were afraid and running all the time;”* and *“[we were] living in a vulnerable situation, running from place to place, always afraid, seeing people arrested and killed, scared and hiding”.*

Several participants described the ability of Burmese soldiers to destroy their entire wellbeing. For example, *“If I don’t work [the Burmese soldiers] will kill my mother [and all of my family and animals];” “The Burmese took everything away, whatever they wanted, and they would beat us if we protested;”* and *“[Burmese soldiers] took away everything on our farm, everything we had”*.

Karen participants over 50 years old offered vivid descriptions of having lived in a state of fear and oppression for most of their lives. One participant stated that her persecution started when she was six years old, describing, *“I saw so many things happen when we were running - mortar shells, bullets coming down like rain…”* Other participants described, “*Every time I heard the Burmese were coming I ran. I am the person who was scared all the time*” and “*My whole life, I was running away all the time. Run away, build a new house, [Burmese soldiers] come and take everything and destroy it and we have to move again…all my life”.* One participant explained, *“I never saw peace my whole life”.*

#### Systematic destruction/burning of house or village

The systematic destruction and burning of houses and villages also seems to be particularly characteristic of Karen refugees’ experiences. It was described as a strategy widely used by the Burmese military, justifying the creating of a more specific code in addition to the existing HURIDOCS Micro-thesauri codes of *destruction of property* and *arson as a method of indiscriminate attack.* Participants described having their homes and villages burned down repeatedly, for example, *“[Burmese] soldiers came to my village, burned the village…[and] forced everyone to flee;” “[I experienced] lots of miserable things. My village was attacked and burned [by Burmese soldiers]. [My] animals were killed;”* and *“Burmese would burn [my] house and food.”* This category also includes systematic attacks and burning of refugee camps. One participant described:

*I was a little girl when the camp was burned twice [by the Democratic Karen Buddhist Army]. There was also shooting at the camp. Some families had places to escape in Thailand but we did not. A friend and their family hid in the well in the middle of the camp and they all died.*

#### Exposure to dead bodies

Participants described being exposed to dead or severely wounded bodies in the context of conflict and displacement. While fleeing from advancing Burmese soldiers they were not able to assist or attend to the dead or people who needed help. A participant described this experience: *“…when we were running…we had to step over dead bodies. We could not help anyone.”* Another participant described seeing dead bodies in Thailand when her refugee camp was attacked by the Democratic Karen Buddhist Army (DKBA).

#### Orphaned in the context of war

Participants described being orphaned as a result of violence and the effects of prolonged war. One participant reported, *“My parents died when I was young. I lived on my own; I grew up without a village…My father committed suicide and my mother died in childbirth”*. Another participant described, *“My parents died when I was little…My father was murdered by the Burmese. My mother was tortured by the Burmese and died a month later”.*

#### Injury caused by landmine

Participants reported serious and debilitating injuries that resulted from stepping on a landmine, often while they were fleeing, hiding in the jungle, or displaced. Participants also reported amputations as a result of landmine injuries. For example, *“I stepped on a landmine when walking up a hill while [fleeing] in the jungle. My leg was blown off”.*

#### Fear of Thai police or deportation from Thailand

Participants also described feeling unsafe and being afraid of Thai police while living in Thailand, especially if they were outside of the refugee camps. Participants described being fearful of deportation into the custody of Burmese soldiers. A participant described*, “We always had to be afraid of being arrested in Burma, and Thailand too”.* Other participants reported: *“[our lives were] very restricted in the [refugee] camp. We couldn’t go out, [or we would be] arrested by Thai police”* and *“We had no freedom. We were afraid of the Thai police”.* Finally, another participant described,

*I used to get arrested by the Thai border patrol. I had to pay money to get out. I wasn’t physically hurt, but I was very scared. They brought us to Burmese border and threatened to hand us over to soldiers.*

#### Harm or killing in the context of war

This code was created to capture general experiences of violence that did not seem to fit into any existing Micro-thesauri codes. For example, participants described, *“Due to oppression we had to leave”* and *“My [relative] was shot through the chest and I had to carry him away. We barely escaped”.* We also used this code when we did not have enough information to determine the circumstances of a traumatic experience. For example, as part of her response, a secondary war trauma survivor reported, *“My brother was killed in the farm fields”.*

### Primary and secondary torture experiences

Participants described the purposeful nature of torture and reported 35 unique types of torture. Five resulted in death and were reported by secondary survivors. Five torture codes were created to describe experiences not captured by existing HURIDOCS Micro-thesauri codes. These include: *forced portering; forced to be a human landmine sweep; forced to be a soldier, including child soldier; forced contact with a dead body; and removal of the eyes.* Forced portering was the most widely reported of the additional codes that were created.

**Table 2:** **Types of torture reported by primary and secondary survivors**

**Type**	**Primary survivors**	**Secondary survivors**
Beating	x	x
Slapping, kicking or punching	x	x
Blows with rifle butts, whips, straps or heavy sticks	x	x
Wounding	x	x
Maiming or breaking bones	x	x
Being bound or tied up as a form of immobilization	x	x
Arrest and detention/imprisonment	x	x
Stress to the senses	x	x
Exposure to extreme hot or cold	x	x
Deprivation of food and water	x	x
Deprivation of needed medical attention	x	x
Involuntary or forced labor	x	x
Rape	x	x
Psychological torture and ill-treatment	x	x
Psychological assault, harassment	x	x
Degradation (verbal abuse)	x	x
Death threats (against the victim, victim's family or friends)	x	x
Witnessing the torture of others (family, friends, other prisoners)	x	x
Forced portering^a^	x	x
Forced to be a human landmine sweep^a^	x	x
Mass roundup	x	
Asphixiation (“Submarino,” includes the use of water)	x	
Slavery (forced domestic servitude)	x	
Application of electric shock	x	
Forced contact with a dead body^a^	x	
Forced postures		x
Nakedness as a form of degradation		x
Torture (unspecified)		x
Burning as a method for killing		x
Deliberate killings of specific individuals		x
Deliberate killing of a noncombatant		x
Death in detention or police custody		x
Death as a consequence of torture or brutality		x
Removal of the eyes^a^		x
Forced to be a soldier, including child soldier^a^		x

^a^Codes created in addition to existing HURIDOCS Micro-thesauri codes.

### Purposeful nature torture

In reporting their experiences, participants described the strategic use of torture to instill fear and intimidation. Karen leadership and anyone suspected of supporting the resistance movement were specifically targeted and often tortured publically. Civilian men, women and children were also targeted randomly in order to create a climate of fear and intimidation.

Participants described especially violent and public methods of torture and killing for persons suspected of being a part of or supporting the Karen National Union (KNU), the primary political organization representing Karen people. One participant described, *“One of my younger brothers was involved with Kawthoolei [KNU]. They hunted him and killed him”.* Entire families of suspected KNU supporters were also targeted. For example, *“The Burmese took me away and put me in prison. They tied my wrists and hurt me because they suspected that I was supporting the KNU. They also arrested my infant son and my two other sons”* and *“Burmese killed my uncle and aunt, and my mother was forced to watch that. It was very brutal. They cut their throats and stabbed them. They killed them in a very bad way, not like just with guns”.* Another participant described the torture and public display of her father’s body:

*My father was KNU…he was killed brutally by the Burmese when he was arrested…He was tortured badly. His naked body was put on display in the village. No one could bury him. When [my mother and I] escaped, the Burmese soldiers killed my uncle and my father’s [bodyguard] brutally.*

One participant described that it was impossible to defend herself against accusations of supporting the KNU: *“We had no rights in our village. Burmese soldiers came and asked for information about the Karen army. If you say yes or no [that you have information or you don’t] they punish you”.*

Civilians were also randomly targeted for torture. For instance, participants reported the public torture of pregnant women, described by one participant in this way: *“When I was pregnant I was arrested and kept in the sun all day…I was arrested and punished”.* Sometimes individuals were severely tortured, to the point of near-death, and then released back into the community to perpetuate shame and fear.

Torture involved physical as well as psychological pain and suffering, such as being forced to witness the torture of family members or others. A participant described the experiences of his family in this way:

*The Burmese arrested my father. He almost died and barely survived. They came to our village for no reason and arrested him…The Burmese killed my grandfather and uncle in front of my father. The village headman rescued my father [and they allowed him to be released]…Another uncle is facing the same thing in Burma right now.*

Another participant described the random targeting of her son and the debilitating physical effects of his torture, “*My one son, when he was [an adolescent], he didn’t do anything wrong, but he was arrested [by Burmese soldiers] and beaten. [Now he is physically disabled]. We were treated so badly”.*

Participants described the public torture and humiliation of entire villages in these ways: *“My brother and the whole village was arrested and tortured. They put all the women on the ground and tortured all the men”* and *“[Burmese soldiers] forced [the whole village] to do labor, took off their clothes, tied their hands, and put them in the sun all day with no food or clothes”.*

#### Forced portering

*Forced portering,* often referred to by participants as “donation labor”, describes being forced to carry heavy machinery, ammunition, or other supplies for the Burmese military. This experience was commonly reported in this study, justifying creation of a more specific code, *forced portering,* in addition to the existing HURIDOCS Micro-thesauri code for *involuntary or forced labor,* which was also frequently reported. Forced portering often involved severe physical pain and suffering, such as carrying very heavy weaponry that caused permanent back, neck and shoulder injuries, being unable to put the weapons or material down at the point of pain, and being beaten if they had to stop. Participants described forced portering in these ways: *“I was a forced porter… beaten severely, forced to climb high mountains;” “I was forced to carry ammo for soldiers. I saw people beaten badly. The [Burmese] soldiers were yelling at me, shot at me, and threatened me;”* and *“While I was a forced porter I was kicked constantly because I couldn’t keep up”.*

Participants described conditions forced porters experienced as being so severe that some porters died. This included deliberate killing of porters, death as a consequence of portering, and death after being released as a result of the injuries sustained in the context of portering. Porters were denied food, water, and needed medical attention, and were left in the jungle when they became too sick or injured to continue. For example: *“My father was killed as a forced porter. Burmese soldiers didn’t feed him. He got so sick and tired that they left him. He tried to get home but one week after he got home he died;”* and, *“My husband was forced to porter so many times. He was kicked and beaten and eventually he got very dizzy, weak, and died”.* Another participant reported that his brother was denied needed medical treatment and was tortured nearly to death in the context of portering: *“My brother got malaria when he was a forced porter. He couldn’t work for [Burmese soldiers] anymore and they beat him badly….he was tortured so badly he almost died”.*

Psychological torture in the context of forced portering involved witnessing or being forced to witness the torture of others and severe humiliation. One participant reported, *“My parents were forced porters…they had to carry all day and night and were kicked by [Burmese] soldiers. They killed my father’s friend in front of him. He is still affected by this today”.* Participants also described being dehumanized and treated like slaves or animals. For example, *“It was slavery. I was forced to porter countless times”.* In reporting his experience of being forced to porter as a child, another participant described, *“I didn’t even know the value of myself until I got [to the U.S.]. They treated us like animals”.*

Men, women, and children were all forced to porter, and many participants reported being forced to porter many times. One participant reported that her village was forced to provide porters to the Burmese military on a regular basis. When her husband was not available, she had to fill in his place: “*My husband did it, and when he was gone I had to do it… I cannot refuse because they would put you in jail”.*

#### Forced to be a human landmine sweep

*Forced to be a human landmine sweep* describes the experience of being ordered to walk ahead of the advancing military in an area suspected of having landmines, or to “clean landmines”. Human landmine sweeps were used to detonate or remove any landmines on the path so that Burmese soldiers could travel more safely and were also used during active fighting. One participant described multiple, severe, and debilitating torture that included being forced to be a landmine sweep. He described his experiences as: “*forced to be a mine sweep, forced labor, imprisonment, forced to stay out in the elements, and electric shocks. I couldn’t walk for three years.”* He also reported, *“my father died as a mine sweep”.*

Another participant recalled,

*I was captured in jungle and brought to outpost where they asked me to clean landmines. I was wounded by bullets and mortar and got shrapnel in my back and neck from the battle. I passed out from injuries from shrapnel. The military threw my body in the valley. Karen National Union soldiers found me and helped me.*

Participants also reported being forced to do landmine sweeping while they were also being forced to porter. One participant described these experiences from his childhood: *“Burmese soldiers forced me to do portering…I had to carry heavy things and do landmine sweeping”.*

#### Forced to be a soldier, including child soldier

Participants reported that both adults and children were forced to serve as soldiers for the Burmese military. One participant reported that Burmese soldiers systematically targeted the children in her village and forced them to be soldiers and porters. She described, *“The Burmese soldiers asked 14-16 year olds to be soldiers and carry weapons. They were all taken”.*

#### Forced contact with a dead body

Participants described being forced to have contact with dead bodies as a method of torture. One participant described being forced to bury the dead body of a relative who had been burned alive, after several of his family members were also brutally killed. Another participant described the severe and public torture of her father and public display of his naked body, which she was prohibited from burying. From participants’ descriptions, it appears that Burmese soldiers used dead and sometimes mutilated bodies of people suspected of being affiliated with the Karen resistance in order to terrify and intimidate. For example, along with other torture experiences, a participant described being forced to sleep next to the body of a dead Karen soldier: “*A [Karen] soldier was killed by my house, and I had to sleep by the dead body”.*

#### Removal of the eyes

One secondary survivor reported that Burmese soldiers tortured her uncle by taking out his eyes before killing him.

### Torture and war trauma experienced as children

Although this study did not interview people under the age of 18, participants recalled experiencing torture and war trauma as children. The most commonly reported childhood experiences of torture were forced portering and forced labor. Participants described, *“I was a forced porter when I was [an adolescent]. I witnessed fighting and people being beaten, killed, and raped”* and *“Since I was [a child] I was forced to work by Burmese soldiers”.*

One participant recalled being tortured as a child while he was tending his livestock:

*In Burma, when I was a little boy I was taking care of my [livestock] in the jungle. Burmese soldiers took me away. They threatened to cut my throat. I was crying. Some Karen porters saw me and stopped them. They let me go. They were just playing with me.*

Another participant described that his aunt and her children were targeted as a family, “*My aunt was beaten to death with her kids, maybe raped, [and] tortured*”.

Young adult participants described various scenes from growing up in the context of protracted war, including witnessing the torture, rape, and abuse of family members, friends, and other villagers; living in constant fear; multiple forced displacements; and direct actions which violate the right to education. For example, *“I was young, but I heard and saw all the time things happening around my house and village. Burmese soldiers came, raped women, forced labor. We had to leave”, “When I was a little boy Burmese soldiers forced me to carry water. Then they burned down my house and they burned my school”,* and *“When I was young…and going to school, the Burmese military took me from school and forced me to porter…they would hurt us badly [by beating]…They also took my dad and I saw them hit him”.*

## Discussion

Karen people have endured the world’s longest, ongoing civil war [44]. They are currently one of the largest refugee groups being resettled to the United States and other western countries [4]. In response to CDC recommendations for mental health screening, this article examined whether Karen refugees would report war trauma and torture experiences and what they would report in the context of an initial public health screening. The findings provide research-based evidence that Karen people are motivated to report traumatic experiences that may impact their health. The experiences described in this study also confirm human rights reports of widespread and systematic abuses against Karen men, women and children, including the specific use of forced portering and forced labor [20]. Our analysis suggests that additional categories of human rights violations be added to existing documentation systems, including the HURIDOCS Micro-thesauri system, which is widely used by torture treatment centers.

Many refugees have never received adequate healthcare, and the initial public health screening is their first opportunity to have health and mental health needs identified, especially those that may affect their ability to resettle successfully. The initial health screening presents an opportunity for mental health screening because refugees are often motivated to obtain health care when first resettling. For example, although public health screening is voluntary and compliance varies from state to state, one study of 9 large resettlement sites reported that 76% of refugees attended the initial public health screening [45]. In the state where this study was completed, 99% of refugees attend the initial public health screening. Additionally, refugees identified with mental health symptoms may access treatment in their first 8 months of resettlement through refugee medical assistance.

Research also indicates that physicians are reluctant to ask about traumatic histories. One of the concerns raised by these physicians is whether refugees can talk about these experiences in the context of time-limited primary care appointments [46,47]. This study demonstrated that Karen participants were able to talk about their trauma histories with trained interviewers and interpreters and contain these discussions within the short time provided for mental health screening. Furthermore, these findings confirm our previous research finding that refugees are motivated to talk about their traumatic experiences when it may affect their health. Refugees also want health providers to understand the political context of their trauma, validate their experiences, and provide education about the impact of trauma on health as well as treatment options [46]. Due to a lack of knowledge about mental health and treatment options, shame, mistrust, or cultural norms that value deference to authority, refugees may not initiate the conversation and prefer that providers inquire about these experiences [46,48]. For example, a study participant reported that she had been shot in the head while fleeing a village but had never reported this injury to any other health provider because no one had inquired about her experiences of political violence.

Physicians in this study reported that it is helpful to be aware of patient’s traumatic experiences, especially prior to conducting invasive examinations and procedures [8]. Conducting examinations without establishing trust can re-traumatize or trigger a patient because patients may not entirely distinguish between their history of authority figures exercising power to torture and a medical authority conducting an unknown and unexplained procedure. When physicians validate the traumatic nature of torture experiences, they are also establishing trust needed to proceed with physical examinations. When physicians fail to uncover the extent of traumatic experiences, they may be unable to fully evaluate the physical and mental health care needs of refugee trauma survivors and fail to initiate needed treatment or referrals.

Providers who work with refugees may need training on how to create a safe environment, build trust, and ask empathically about traumatic experiences [46-48]. Several scholars have documented effective ways for providers to ask survivors directly about their trauma histories without retraumatizing [6,48]. Consistent with CDC recommendations, we also believe having knowledge of the sociopolitical history and traumatic experiences commonly reported by Karen people provides context for understanding individual patient’s stories. Understanding these experiences is also necessary for providing mental health care. For example, some evidence based practices for treating trauma involve exposure to the detailed narratives of traumatic experiences [49].

Our findings from interviewing refugees directly about their experiences support the additional categories that Cardozo and colleagues added to adapt the HTQ for Karenni refugees based on key informant interviews [24]. They also indicate the usefulness of the HURIDOCS Micro-thesauri coding system as a valuable tool for documenting human rights violations. Codes that were created in addition to existing Micro-thesauri codes add to general international knowledge of human rights violations and of the experiences that are more characteristic of the Karen context. New war trauma codes include: *widespread community fear, systematic destruction/burning of house or village, exposure to dead bodies, orphaned in the context of war, injury caused by landmine, fear of Thai police or deportation from Thailand, and harm or killings in the context of war*. New codes created to capture additional torture experiences reported include: *forced portering; forced to be a human landmine sweep; forced to be a soldier, including child soldier; forced contact with a dead body; and removal of the eyes*. Lastly, the study demonstrates the strengths of using qualitative methods to uncover broader knowledge about the experiences of Karen refugees.

The documentation of widespread use of forced porters by the Burmese military is particularly relevant given recent ceasefire negotiations between the Burmese government and armed Karen groups. According to a recent report, practices such as forced labor, land confiscation, and arbitrary taxation are ongoing and may even increase during ceasefires, especially near areas the government is seeking to develop [1]. This report, and Mullany and colleagues’ finding that forced portering was more prevalent in ceasefire regions than in non-ceasefire regions, support the importance of continued monitoring of human rights violations in Burma during times of ceasefires [22].

### Limitations

The brief nature of the refugee health screening naturally limited the extent of information that could be gathered. This study did not ask participants to systematically report all types of war trauma and torture they experienced and does not report frequencies of different types. In particular, participants may have chosen not to report rape or other sexual violence, even if it was experienced, due to the high level of stigma associated with these experiences [18]. This project’s cultural consultant confirmed that “the pain of women” caused by war is often missing in research and human rights reports on the Karen. In our study, rape was almost exclusively reported by secondary survivors. This study likely did not capture the extent to which rape was experienced by female participants, or the violence and brutality that may have been associated with rape that was reported. Additionally, it is possible that torture experiences are underreported or only reported by secondary survivors due to factors such as shame or the impact of posttraumatic stress, specifically avoidance symptoms and lack of verbal memory [40,48]. Despite these limitations, this paper adds understanding of a broader range of experiences than had been previously reported in research.

There were two additional events that emerged from participants’ responses for which we did not have enough data to justify creation of new codes. One participant described being forced to work as an interpreter (Burmese-Karen) for Burmese soldiers to support their military operations near her village. Although she reported it as one of many traumatic experiences, we did not elicit enough detail about the act of being forced to interpret to determine whether it, in itself, caused severe physical or psychological suffering necessary to constitute torture. From another participant’s description, it is possible that Burmese soldiers may have used him as a human shield in combat. The use of Karen people as human shields has been previously documented [50]. However, we did not elicit enough detail from this participant to be able to determine whether he was intentionally used as a human shield. Nonetheless, we feel these experiences are notable and warrant further consideration in future research.

## Conclusions

The recently released CDC recommendations for routine mental health screening of newly arriving refugees include providers being knowledgeable about the histories of the groups they serve and inquiring about patients’ exposure to trauma. This article reports the traumatic experiences that Karen refugees were willing and able to describe in the context of a short screening. All Karen refugees who identified as being war trauma and/or torture survivors were able to describe at least one event that may impact their health or mental health in resettlement. In addition, this study demonstrates the usefulness of the HURIDOCS Micro-thesauri coding system used by many torture treatment centers and suggests additional codes related to the torture and war trauma experiences of Karen people. Given the high rates of war trauma and torture survivors in this refugee group, health professionals should be familiar with trauma histories to inform their diagnosis and treatment. Physicians may need training to learn how to establish trust and inquire about trauma and the health effects of these experiences in the short time frame of an initial public health screening.

More research is needed to understand the health and mental health effects of the experiences that Karen people reported in this study, for example, the physical health consequences of forced portering. In particular, research is needed to understand how these experiences affect refugees’ ability to resettle successfully. Since rape is a widespread trauma that is underreported in this study, more research is also needed to understand how to talk to Karen women about these experiences to facilitate access to treatment when necessary.
